# The effects of sericin on cryopreserved sperm cells and subsequent embryo development in mice

**Published:** 2018-06

**Authors:** Mona Ghasemi, Abbas Farshad, Hadi Hajarian, Omid Banafshi, Vahideh Asadollahi, Fardin Fathi

**Affiliations:** 1 *Department of Animal Sciences, Faculty of Agriculture, University of Kurdistan, Sanandaj, Iran.*; 2 *Department of Animal Sciences, Razi University, Kermanshah, Iran.*; 3 *Cellular and Molecular Research Center, Kurdistan University of Medical Sciences, Sanandaj, Iran.*

**Keywords:** Cryopreservation, In vitro fertilization, Mouse, Sericin, Sperm

## Abstract

**Background:**

Sericin, because of its ability to remove free radicals and its antioxidant properties, has been used to successfully cryopreserve various mammalian cell types. However, the effects of sericin on cryopreservation of mouse sperm has not been reported.

**Objective:**

The current study intended to determine the protective role of different concentrations of sericin (0, 0.25, 0.5, and 0.75%) on mouse spermatozoa during cryopreservation, in addition to its effect on in vitro fertilization and subsequent embryo development.

**Materials and Methods:**

Mouse sperm from epididymides were frozen in cryoprotective agent with 18% raffinose, 3% skim milk, and different concentrations of sericin (0, 0.25, 0.5, 0.75%). Thawed sperm were used for in vitro fertilization. The obtained embryos were cultured in Ksom medium for 6 days. The post-thawed motility, viability, fertilizing ability, and subsequent development to the 2-cell embryo and blastocyst stages were evaluated.

**Results:**

Our findings show that frozen-thawed sperm cells with 5% sericin indicate significantly (p≤0.0001) percentages of survivability and motility, the best fertilizing ability, as well as 2-cell embryo and blastocyst development compared to the other treated groups. There was no significant difference in survivability (p=0.8781), fertilizing ability (p=0.2458) and development of 2-cell (p=0.5136) and blastocysts embryos (p=0.0896) between 0.75% sericin and control groups.

**Conclusion:**

Supplementation by 0.5% sericin in cryoprotective agent improved frozen-thawed mouse epididymal sperm cell quality and resulted in increased embryo development**.**

## Introduction

Sperm freezing is a critical method for long-term sperm storage, but irreversible damage to sperm membranes is partially induced by cryopreservation (1, 2) which may reduce sperm motility, viability, and fertilization rate following insemination (3). Successful cryopreservation of murine sperm has been reported in the 1990s (4). 

Further developments in sperm freezing methods show increased success (5). Improved fertilization can be achieved using sperm cryopreserved in a solution of 18% (w/v) raffinose and 3% (w/v) skim milk. Therefore, identification of a suitable cryoprotectant is necessary in to minimize loss because of cryo-damage and improve freezability of mouse spermatozoa. According to the researches, sericin suppresses lipid peroxidation (LPO) (6), prevents cell death (7), protects from freezing (8, 9), and protects sperm from oxidative stress (10). It is a supplement for IVM in bovine oocytes (11) and bovine embryos (12). 

Sasaki and co-workers report that serum-free mammalian cell culture, could recover whit sericin particularly sericin S (9). Dash and co-workers have stated that sericin can be used as a worthwhile antioxidant (13). According to Isob and colleagues sericin can prohibit oxidative stress at the time of bovine embryo culture with increased embryo quality and improved embryonic development (14). Do and co-workers Reported that addition of 1.0% sericin at the time of the maturation culture resulted in negligible effect on porcine oocyte maturation, fertilization and embryo quality, but the formation of blastocyst was not affected (15). Replacement of fetal bovine serum by sericin in maturation culture medium has been shown to expand the perivitelline space, increase hyaluronic acid secretion, and decrease polyspermic fertilization in bovine oocytes (11). 

Yasmin and colleagues reported that 0.1% sericin addition improved maturation and total fertilization rates of sheep oocytes cultured in a maturation culture medium containing serine without bovine serum albumin (16). Hajarian and colleagues showed that sericin could be a substitutive protein supplement for in vitro maturation and in vitro culture of ovine zygotes and oocytes (17). In a recent review, numerous reports showed that sericin could be an additive or a novel serum substitute for cell culture and cryopreservation media (18).

However, we located no report that used sericin in freezing media in an attempt to lessen the damages to sperm during cryopreservation process. As a result, the current study intended to examine various concentrations of the silk protein sericin in semen cryopreservation media to improve freezability and increase the blastocyst rate in mouse spermatozoa.

## Materials and methods

**Sericin**

Sericin was purchased from Sigma-Aldrich Chemicals Pvt. Limited, Cat no. S5201.

**Media**

Modified 18% raffinose pentahydrate and 3% skimed milk solution (DifcoTM, Beckton Dickinson and Co., Franklin Lakes, NJ, USA) were used for sperm cryopreservation (19). Human tubal fluid medium (HTF, Millipore) was used for IVF (19, 20) and potassium simplex optimization medium (KSOM, Millipore) for the subsequent development of 2-cell embryos to the blastocyst stage (19).

**Mouse sperm cryopreservation and thawing: Preparation of cryoprotective agent (CPA)**

CPA was made by pouring 8 ml of MQ into one 15 ml falcon tube, followed by the addition of 1.8 gr of raffinose. The tube was placed in 80ºC water bath for 30 sec. The solution was dissolved by gentle inversion. Next, we added 0.3 gr of skimed milk and gently mixed the solution. The final 10 ml volume was centrifuged at 10000×g for 30 min at room temperature. The clear supernatant was filtered through a 0.22-μm filter and stored in aliquots at 4ºC for up to 10 days (19).

**Sperm collection, processing, and freezing**

In total 130 μl of CPA was transferred to a 35-mm sterile plastic tissue culture dish and covered with mineral oil. A male NMRI mouse was selected and killed by cervical dislocation. Next, we transferred two cauda epididymides from this mouse to a dish that contained CPA. Then a 5-6 incisions was made in the epididymides with a pair of sharp-pointed forceps and micro-spring scissors, then the dish was carefully rotated to ensure uniform sperm suspension. After 3 minutes we removed the cauda epididymides from the dish. In order to assess sperm quality, 1 μl of the sperm suspension was transferred into 100 μl of HTF medium and covered with mineral oil in a dish. The dish was allowed to incubate for 10 min to ensure adequate dispersion of the sperm suspension in HTF. 

Prior to freezing, we subjectively assessed both the motility and viability level of the collected spermatozoa under phase contrast microscope (400× magnification). Only ejaculates that had ≥70% sperm motility were cryopreserved. The pooled sperm were allocated into the following groups of CPA supplemented with various concentrations of sericin: 0 (control), 0.25%, 0.5%, and 0.75%. A 1-ml syringe was connected to a 0.25-ml plastic straw using a silicon tube where 100 μl of HTF medium, 10 mm of air, and 10 μl of the sperm suspension were carefully aspirated into the straw in succession, followed by an additional 10 mm of air. The end of the straw was sealed with PVA by pushing the end that contained 10 μl of medium into the powder. The straws were placed in liquid nitrogen vapor at -150ºC. After 10 min, the straws were plunged into liquid nitrogen at -196ºC.

**Thawing**

The frozen straw was removed from the liquid nitrogen and put in the air for 5 sec, after which it was rapidly placed in a 37ºC water bath for 10 min. The thawed sperm was transferred to a dish that contained 100 μl of preincubation HTF medium. The dish was placed in an incubator for 30 min.

**Assessment of sperm motility and survival**

We assessed the motility and survival of the frozen-thawed sperm at 0 and 24 hr by light microscope at 400x magnification. The proportion of progressive or not progressive sperm cells were counted. Supravital staining was used to evaluate sperm survival. The sperm suspension drop was placed on a spot plate. One drop of a 1% aqueous eosin solution was added to this drop and mixed together. After 25 sec, the solution absolutely mixed with two drops of 10% aqueous nigrosin solution. A thin smear was made and allowed to air dry. Dead sperm stained pink whereas live sperm cells were white. We counted approximately 300 spermatozoa and expressed the result as; Live sperm percentage.

**In vitro fertilization using cryopreserved spermatozoa**

Superovulation was induced in female NMRI mice with 5 IU pregnant mare serum gonadotropin (PMSG, Sigma) injection, followed by 5 IU human chorionic gonadotropin (hCG, Sigma) injection 48 hr later. Mice were sacrificed by cervical dislocation 12-13 hr after administration of hCG; their oviducts were immediately removed and placed in fertilization dish that contained paraffin oil. The cumulus-oocyte complexes (COCs) were placed in a second dish that contained a 100 μl drop of HTF medium, covered with paraffin oil. 10 μl of sperm was added to every drop of fertilizing HTF medium. 

The obtained oocytes were incubated with spermatozoa for 4-6 hr. Subsequently, we washed the oocytes in order to eliminate extra spermatozoa. The number of one-cell embryos were recorded and then cultured overnight in separate dishes, in a drop of KSOM. The next day, we placed the obtained 2-cell embryos in KSOM covered by mineral oil at 37ºC in an atmosphere of 5% CO_2_ and air. These embryos were allowed to develop for four days until the blastocyst formation. Their developmental stages were defined by morphological evaluations conducted every 24 hr under a stereomicroscope. Fertilization rate was determined as the rate of 2-cell embryos observed 24 hr after insemination.

**Ethical consideration**

All mice were used according to the Guidelines for the Care and Use of Laboratory Animals by Kurdistan University of Medical Sciences and has been approved by Kurdistan University of Medical Sciences Ethical Committee (Code no. 34099). 

**Statistical analysis**

A 4×2 factorial design along with a positive control outside the factorial formed the design of this experiment. The data analyzing was done with ANOVA by using of the GLM procedure (2003). The treatment means were compared with the Bonferroni least significant difference procedure. Significant differences among the factoria and the means of positive control treatment were checked by Dunnett’s test. p<0.05 was considered statistically significant. 

## Results

We observed that addition of sericin to the sperm media used for cryopreservation influenced sperm survivability, motility, fertilizing ability, and subsequent development to 2-cell and blastocyst embryos after the freeze-thaw process. We observed significantly (p≤0.0001) highest percentages of survivability and motility in the 0.5% sericin supplemented medium compared to the other groups (Table I). A significant decrease (p≤0.0001) existed in survivability and motility rate at 24 hr after freezing in 0% group in comparison to the control and 0.5% sericin groups. No significant difference (p=0.3628) existed in survivability between the 0.75% sericin (49.20±1.54%) and control (51.25±2.74%) groups at 0 hr of storage post-thaw. Table II shows the effects of the sericin concentrations and post-thaw storage time on sperm fertilizing ability and subsequent development to the 2-cell and blastocyst embryos. 

Our results showed significantly greater rate of sperm fertilizing ability in the 0.5% (58.52±12.12%, p≤0.0001) and 0.25% (54.41±10.93%, p=0.0018) sericin groups compared to the control group (46.06±11.64%). However, we observed the highest rate in the 0.5% sericin group. There was no significant difference must be replaced with significantly no differences in fertilizing ability (p=0.2458) and development of 2-cell (p=0.5136) and blastocysts embryos (p=0.0896) between the 0.75% sericin and controlgroups. After 6 days of culture we observed significantly (p≤0.0001) greater percentage of blastocysts in the 0.5% sericin group (50.52±9.92%) compared to the control (36.46±7.12%).

**Table I:** Effects of different concentration of sericin (0%, 0.25%, 0.5%, and 0.75%) and post-thaw storage times on sperm survivability and motility

**Sericin (%)**		**Time (hr)**	**Survivability (%)**	**Motility (%)**
Positive control				
	––	––	93.93 ± 1.26	86.80 ± 0.39
	0	0	51.25 ± 2.74 c*	42.65 ± 2.02^c^*
	0	24	27.47 ± 1.43^f^*	20.41 ± 1.51^g^*
	0.25	0	54.88 ± 2.35^b^*	48.67 ± 2.17^b^*
	0.25	24	34.01 ± 1.85^e^*	26.26 ± 1.66^e^*
	0.5	0	60.24 ± 0.82^a^*	52.56 ± 0.74^a^*
	0.5	24	40.44 ± 0.58^d^*	33.91 ± 0.74^d^*
	0.75	0	49.20 ± 1.54^c^*	42.40 ± 0.74 c*
	0.75	24	31.89 ± 0.82^e^*	24.26 ± 0.73^f^*
Main effects				
	0		39.36 ± 12.70^c^	31.53 ± 11.84^d^
	0.25		44.45 ± 11.18^b^	37.46 ± 11.95^b^
	0.5		50.34 ± 10.45^a^	43.24 ± 9.85^a^
	0.75		40.55 ± 9.19^c^	33.33 ± 9.58^c^
	Time (hr)			
	0		53.89 ± 4.67 a	46.57 ± 4.61^a^
	24		33.45 ± 4.93^b^	26.21 ± 5.17^b^
	Pooled SEM		0.0448	1.946
	Significance			
	Positive control vs. factorial		<0.0001	<0.0001
	Sericin		<0.0001	<0.0001
	Time		<0.0001	<0.0001
	Sericin × time		0.002	0.002

a-g Least squares explain that a column contain different superscripts differ significantly (p<0.05).

* p<0.05: Difference due to positive control by Dunnett’s test. 1 SEM= Standard error of the mean

**Table II:** Effects of sericin concentrations and post-thaw storage time on sperm fertilizing ability and development of 2-cell and blastocysts embryos

**Sericin (%)**		**Time (hr)**	**Fertilizing ability1**	**2-cell embryo2**	**Blastocysts embryo3**
Positive control					
	––	––	85.73 ± 4.05	74.19 ± 1.71	67.87 ± 3.65
	0	0	56.02 ± 3.50*	48.55 ± 3.53*	42.67 ± 3.55*
	0	24	36.10 ± 3.04*	34.22 ± 3.57*	30.25 ± 1.94*
	0.25	0	64.15 ± 3.37*	53.51 ± 3.67*	48.99 ± 4.66*
	0.25	24	44.67 ± 4.52*	41.65 ± 1.97*	35.71 ± 2.78*
	0.5	0	69.07 ± 3.58*	63.91 ± 2.88*	59.13 ± 3.82*
	0.5	24	47.96 ± 6.28*	44.60 ± 4.51*	41.90 ± 4.64*
	0.75	0	59.07 ± 1.45*	51.63 ± 1.77*	44.39 ± 5.16*
	0.75	24	37.74 ± 2.81*	34.84 ± 0.41*	31.23 ± 4.53*
Main effects					
	0		46.06 ± 11.64^c^	41.39 ± 8.80^c^	36.46 ± 7.12^c^
	0.25		54.41 ± 10.93^b^	47.58 ± 6.83^b^	42.35 ± 7.88^b^
	0.5		58.52 ± 12.12^a^	54.26 ± 10.78^a^	50.52 ± 9.92^a^
	0.75		48.41 ± 11.44^c^	43.24 ± 8.93^c^	37.81 ± 8.31^c^
	Time (hr)				
	0		62.08 ± 5.48^a^	54.40 ± 6.30^a^	48.79 ± 7.66^a^
	24		41.62 ± 6.42^b^	38.83 ± 5.33^b^	34.77 ± 5.79^b^
	Pooled (SEM)		2.289	1.939	1.634
	Significance				
	Positive control vs. factorial		<0.0001	<0.0001	<0.0001
	Sericin		<0.0001	<0.0001	<0.0001
	Time		<0.0001	<0.0001	<0.0001
	Sericin × time		0.93	0.06	0.56

1 Fertilizing ability= (no. of fertilized oocytes/total no. of oocytes examined) × 100;

22-cell embryo= (no. of 2-cell stage embryos/total no. of oocytes examined) × 100;

3Blastocysts embryos= (no. of blastocysts embryos/total no. of oocytes examined) × 100; SEM= Standard error of the mean.

a-c Least squares explain that a column contain different superscripts differ significantly (p<0.05).

* p<0.05: Difference due to positive control by Dunnett’s test.

**Figure 1:**
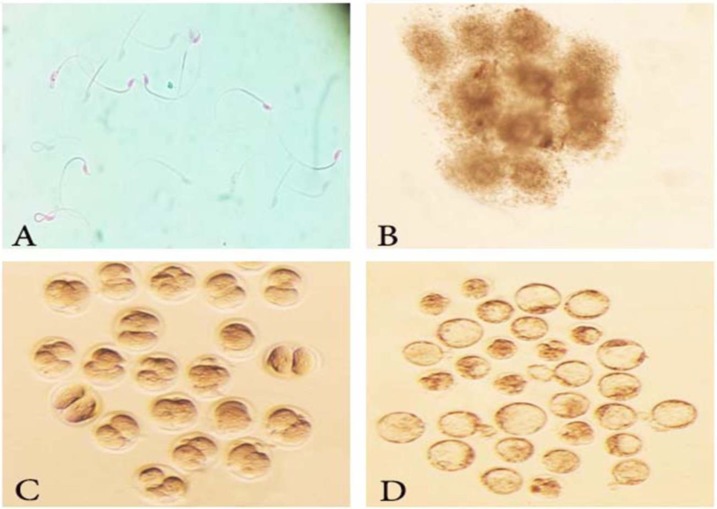
Development of mouse preimplantation embryos fertilized with thawed sperm from cryoprotective agent (CPA) +0.5% sericin. IVF was performed in human tubal fluid (HTF) medium and the resultant two-cell stage embryos were developed to blastocysts in potassium simplex optimization medium (KSOM) . Morphologies of: A) Thawed sperm stained with eosin nigrosin. B) Cumulus-oocyte complexes (COCs) C) Two-cell embryos produced 24 hr after fertilization. D) Morula and blastocysts developed in KSOM medium 6 days after fertilization. (magnification: 40x).

## Discussion

In this study our results for the first time in mouse model showed that supplementation with 0.5% sericin during mouse sperm cryopreservation significantly affected the survivability, viability, fertilizing ability, and subsequent development to 2-cell and blastocyst embryo stages. The goal of sperm cryopreservation is to achieve high numbers of normal sperms that survive after thawing. However, numerous factors during the process of cryopreservation can affect the post-thawing consequence (21). Plasma membrane of Buffalo sperms contain numerous polyunsaturated fatty acids, which makes it greatly susceptible to LPO (22). In conclusion, LPO of the sperm membrane give rise to the deficiency of sperm function due to reactive oxygen species, alterations in membrane integrity, sperm motility and fertility due to oxidative stress (23, 24).

The results of this study where frozen-thawed sperm with 0.25% and 0.5% sericin had higher sperm motility and viability agreed with those of Kumar and colleagues who reported that supplementation of the extender with 0.25% and 0.5% sericin led to increased sperm motility, increased plasma membrane integrity, and decreased LPO in buffalo sperm (10). Their ﬁndings supported results published by Terada and colleagues who showed that 1% sericin had deleterious effects on different mammalian cell lines (25). Therefore, cryopreservation media supplemented with sericin reduced the deleterious effects of LPO and resulted in signiﬁcantly higher post-thaw semen quality. It has been reported that sericin as a supplement serum-free media can increase proliferation of different mammalian cells and protect insect cells which acutely deprived from serum (7). 

In the present study, we observed that the 0.5% sericin group had the best fertilizing ability, 2-cell embryo and subsequent blastocyst development compared to the other groups. Supplementation by 0.5% sericin in cryoprotective diluent increased frozen-thawed sperm quality and the blastocyst rate. It has been shown that the sericin increases the percentage of sheep two-cell embryo development to blastocyst (17). Sericin can be used as a suitable alternative to bovine albumin serum for cultivation of different cells and to increase cell proliferation. (25). The addition of 0.5% sericin to In Vitro clulture medium increased the rate of full cumulus expansion and the number of matured oocytes (26). In a recent study, 0.1% sericin supplementation improved the maturation and fertilization rates of sheep oocytes cultured in culture medium that lacked bovine serum albumin (16). 

Therefore, 0.5% sericin might possibly increase ovine embryos development through activation of oocyte-derived factors while adjusting hyaluronic acid secretion in the time of maturation (26). A number of studies have reported the benefit of sericin in cryopreservation of various mammalian cells. For example, sericin was used to successfully cryopreserve islet cells, which increased the practicality of clinical islet transplantation (27). Freezed human adipose tissue-derived stem/progenitor cells that used sericin have successful function in cell transplantation and regenerative medicine (28). 

Compared to the serum-supplemented media in sericin-based freezing media, successfully cryopreserved in vitro fertilized embryos had no differences in the rate of recipients in terms of pregnancy, stillbirth, abortion and normal calving (12).

## Conclusion

The present study revealed that supplementation of semen extender by 0.5% sericin increased frozen-thawed mouse epididymal sperm cell quality which resulted in increased embryo development.

## Note

***This article extracted from M.Sc. thesis. (Mona Ghasemi)***
